# The relationship of T2WI signal intensity of uterine fibroids and the temperature curve in MR-guided high-intensity focused ultrasound (HIFU) ablation

**DOI:** 10.1186/2050-5736-3-S1-O96

**Published:** 2015-06-30

**Authors:** Jia Liu, Bilgin Keserci, Rong Rong, Juan Wei, Xiaoying Wang

**Affiliations:** 1 Peking University First Hospital, Beijing, China; 2 Philips Healthcare, Seoul, Republic of Korea

## Background/introduction

MR guided high-intensity focused ultrasound (HIFU) ablation is increasingly being used worldwide to treat symptomatic uterine fibroids because of its excellent therapeutic efficacy in controlling symptoms and its excellent safety record. High signal intensity on T2WI MR images of fibroids is firstly considered a factor that induces poor ablation outcomes. Nevertheless, to our HIFU clinical practice, signal intensity on T2WI MR images is not that accurate for determining whether the fibroids are hyper-perfusion or hypo-perfusion because signal intensity on T2WI is just a relative and subjective parameter, which cannot quantify perfusion. It is demonstrated that the temperature curve (i.e. temperature change as a function of time) during HIFU treatments is a relatively accurate manifestation of fibroids’ reaction to sonication. The purpose of this study was to retrospectively study the relationship of T2WI signal intensity of uterine fibroids and the temperature curve in MR guided HIFU.

## Methods

**Figure 1:**
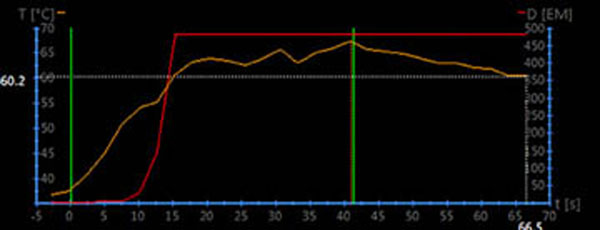
A graph of temperature change as a function of time obtained during treatment in therapy control graphical user interface

**Figure 2:**
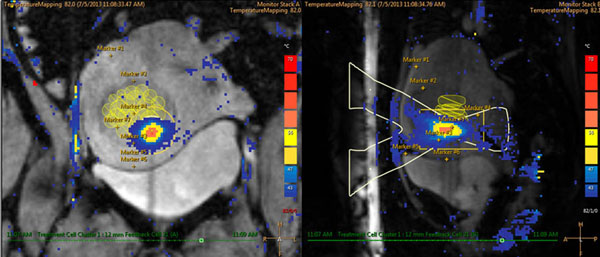
Coronal (left) and sagittal (right) MR thermometry images show temperature overlay during volumetric MR HIFU ablation.

All 15 female patients (mean age, 44.7±5.4 years) who were given written informed consent underwent MRI screening. In the screening, subjects were positioned prone, feet first, on the 3T MRI scanner (Achieva TX, Philips Medical Systems, Best, the Netherlands) with a 32-channel phased array coil. T2W images were acquired on the sagittal plan across the uterus. The fibroids were classified into 3 types according to the signal intensity on T2WI MRI: Type 1(n=7), with signal intensity lower than the skeletal muscle. Type 2(n=7), with signal intensity lower than the myometrium but higher than the skeletal muscle. Type 3(n=1), with signal intensity higher than the myometrium. Thermometry got during the treatments was used to create temperature curves as shown in Figure 1 and 2.

**Figure 3:**
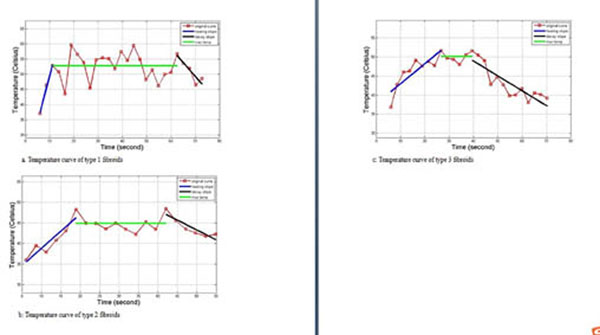
Temperature curve of type 1, 2, 3 fibroids

For better interpretation, two points were chosen on the temperature curve: The first point was the time point at the end of the ascending segment before the temperature got stable. The second point was the time point at the beginning of the descending segment before the temperature began to decay. As shown in Figure 3, the Matlab software accompanied with linear regression and definite integration methods was used to analyze the temperature curve to generate several parameters as follows: (i) *heating slope*, the slope between the beginning point of the curve to the first point; (ii) *decay slope*, the slope between the second point to the last point of the curve; (iii) *area under heating curve* (AUC), the area under the curve between the beginning point of the curve to the second point; (iv) *heating time*, the time span between the first point to the second point of the curve; (v) *max temp*, the average of all temperatures between the first to the second point of the curve; and (vi) *time to peak*, the time span of between the beginning point to the first point of the curve.

## Results and conclusions

As shown in Table 1, the temperature curve of type 1 fibroids ascended quickly (heating slope: 1.67±0.73°C/s, time to peak: 12.5±4.2s), descended quickly (decay slope: 0.83±0.29°C/s) and had the longest plateaus (heating time: 38.5±18.2s). It had the most effective therapy (AUC: 2221.7±769.4°C• s). The temperature curve of type 2 fibroids ascended slowly (heating slope: 0.80±0.66°C/s, time to peak 22.5±11.1s), descended slowly (decay slope: 0.47±0.30°C/s) and had a shorter plateaus (heating time: 24.8±7.2s).

**Table 1:** Parameters of the temperature curves of 3 types of fibroids

Types of fibroids	Heating slope(°C/s)	Decay slope(°C/s)	Heating time(s)	Time to peak(s)	Max time(°C)	AUC(°C • s)
1(n=7)	1.67±0.73	0.83±0.29	38.5±18.2	12.5±4.2	45.3±2.5	2221.7±769.4
2(n=7)	0.80±0.66	0.47±0.30	24.8±7.2	22.5±11.1	45.5±4.1	2036.5±361.5
3(n=1)	0.52	0.39	12.9	20.6	50.1	1597.2

It had a less effective therapy (AUC: 2036.5±361.5°C• s). The temperature curve of type 3 fibroids ascended slowly (heating slope: 0.52°C/s, time to peak: 20.6s), descended most slowly (decay slope: 0.39°C/s) and had the least effective therapy (AUC: 1597.2°C• s, heating time: 12.9s).

The efficacy of HIFU treatments based on temperature curve correlates well with the T2WI signal intensity of uterine fibroids.

